# Role of particle size-dependent copper bioaccumulation-mediated oxidative stress on *Glycine max* (L.) yield parameters with soil-applied copper oxide nanoparticles

**DOI:** 10.1007/s11356-024-33070-x

**Published:** 2024-04-02

**Authors:** Elham Yusefi-Tanha, Sina Fallah, Lok Raj Pokhrel, Ali Rostamnejadi

**Affiliations:** 1 Department of Agronomy, Faculty of Agriculture, Shahrekord University, Shahrekord, Iran; 2 Department of Public Health, The Brody School of Medicine, East Carolina University, Greenville, NC USA; 3 Faculty of Electromagnetics, Malek Ashtar University of Technology, Tehran, Iran

**Keywords:** Copper oxide nanoparticles, Micronutrients, Seed oil, Seed protein, Phytotoxicity, Nanofertilizer, Oil crop

## Abstract

Increased impetus on the application of nano-fertilizers to improve sustainable food production warrants understanding of nanophytotoxicity and its underlying mechanisms before its application could be fully realized. In this study, we evaluated the potential particle size-dependent effects of soil-applied copper oxide nanoparticles (*n*CuO) on crop yield and quality attributes (photosynthetic pigments, seed yield and nutrient quality, seed protein, and seed oil), including root and seed Cu bioaccumulation and a suite of oxidative stress biomarkers, in soybean (*Glycine max* L.) grown in field environment. We synthesized three distinct sized (25 nm = S [small], 50 nm = M [medium], and 250 nm = L [large]) *n*CuO with same surface charge and compared with soluble Cu^2+^ ions (CuCl_2_) and water-only controls. Results showed particle size-dependent effects of *n*CuO on the photosynthetic pigments (Chla and Chlb), seed yield, potassium and phosphorus accumulation in seed, and protein and oil yields, with *n*CuO-S showing higher inhibitory effects. Further, increased root and seed Cu bioaccumulation led to concomitant increase in oxidative stress (H_2_O_2_, MDA), and as a response, several antioxidants (SOD, CAT, POX, and APX) increased proportionally, with *n*CuO treatments including Cu^2+^ ion treatment. These results are corroborated with TEM ultrastructure analysis showing altered seed oil bodies and protein storage vacuoles with *n*CuO-S treatment compared to control. Taken together, we propose particle size-dependent Cu bioaccumulation-mediated oxidative stress as a mechanism of nCuO toxicity. Future research investigating the potential fate of varied size *n*CuO, with a focus on speciation at the soil-root interface, within the root, and edible parts such as seed, will guide health risk assessment of *n*CuO.

**Graphical Abstract:**

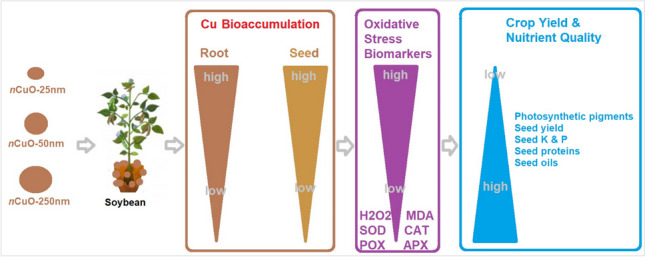

**Supplementary Information:**

The online version contains supplementary material available at 10.1007/s11356-024-33070-x.

## Introduction

Rising human populations combined with global warming and depleting natural sources and arable land exert far-reaching consequences to global food production and food security (Li et al. 2021; Rani et al. 2023). By 2050, the world population is projected to exceed 9.8 billion, necessitating an increase of 50–70% in production to meet the growing food demand (Kusiak et al. 2023; Rani et al. 2023). To ensure food security, it is crucial to make agriculture more sustainable and productive (FAO 2017). The common strategy to improve agriculture production is through application of excess agrochemicals (Li et al. 2021; Hasegawa et al. 2018). However, conventional fertilizers are limited by low efficiency (often below 30%), leading to environmental degradation and significantly polluting air, water, and soil (Hasegawa et al. 2018; Saia et al. 2021; Guo et al. 2021; Deng et al. 2022). Evolving research suggests that nanotechnology may have the potential to promote crop productivity, sustainability, and global food security (Marchiol et al. 2020; Jia et al. 2022; Dilnawaz et al. 2023). Utilizing nano-based agrochemicals may help minimize the use quantity, thereby reducing the environmental burden of agrochemicals and promoting crop nutrient quality and yield through targeted low-dose use and slow release (Deng et al. 2022; Tripathi et al. 2023).

Insufficient animal product consumption for protein needs prompts the importance of incorporating plant proteins like soybean (Taghizadeh et al. 2007; Sudha et al. 2022). Soybean (*Glycine max* [L.] Merr.) is a vital legume with high vitamin, mineral, fiber, and macronutrient content (Sudha et al. 2022). It ranks as the fifth global crop, contributing 40% of yearly oilseed production (Priester et al. 2012), and plays a vital role in global food security (Van Ittersum et al. 2013). With its protein (35–40%), oil (20%), and carbohydrate (35%) composition, soybean is a valuable resource for enhancing human nutrition (Kumar et al. 2017; Lee et al. 2019; Xu et al. 2020).

Copper (Cu) is an essential micronutrient for plant growth and is involved in various physiological processes (Rahman and Schoenau 2020). However, both excess and deficiency of Cu can be detrimental to plants, animals, and humans (Bona et al. 2007; Chandra et al. 2014). Excess Cu can lead to reduced photosynthetic activity, chlorosis, increased disease susceptibility, and stunted growth due to its limited availability and mobility in soil (Rahman and Schoenau 2020; Kusiak et al. 2023). It can also generate reactive oxygen species, leading to DNA damage, oxidative stress, and lipid peroxidation (Nair and Chung 2014; Angel´e-Martínez et al. 2017). Additionally, excess Cu can impact the production of biomolecules such as carbohydrates, proteins, lipids, fatty acids, and photosynthetic pigments such as chlorophyll a (Rocha et al. 2021). These effects are similar to those observed in Cu deficiency, including reduced electron flow, quantum yield, and growth rate (Rocha et al. 2021). Copper-containing fertilizers, fungicides, and bactericides have been used extensively in modern agriculture (Sonmez et al. 2006). Copper chloride, for instance, aids in the enhancement and sustenance of crop productivity owing to Cu bioavailability (Apodaca et al. 2017). Most recently, synthetic nanoparticles (NPs) with unique properties, such as higher adsorption and slow-release potential, have garnered increased interest in agriculture (Rajput et al. 2018; Xiao et al. 2022; Jia et al. 2022).

Metal-based nanoparticles (MNPs), particularly copper oxide nanoparticles (*n*CuO), are commonly employed in seed coatings, pesticides, fungicides, herbicides, and fertilizers to enhance crop production (Dimkpa et al. 2019; Wang et al. 2020; Shang et al. 2021; Xiao et al. 2022). However, the effects of nCuO on plants can be both positive and negative, depending on dose and particle size (Hofmann et al. 2020; Deng et al. 2020; Xiao et al. 2022). Excessive *n*CuO application has shown adverse effects on plant biomass and nutrient content, while optimal amounts can promote nutrient transport and crop nutritional value (Wang et al. 2020; Pelegrino et al. 2021; Kusiak et al. 2023). Crops are known to transport and bioaccumulate MNPs in edible parts, raising concern for potential human health risks (Deng et al. 2020; Bajaj et al. 2023). Therefore, it is important to investigate the potential effects of MNPs on the edible parts of crops (Rui et al. 2018; Yusefi-Tanha et al. 2020a, b, 2023). Studies evaluating the effects of *n*CuO on yield and seed nutritional quality after full life cycle exposure under field conditions are scarce (Wang et al. 2021). In light of the above, it was hypothesized that Cu bioaccumulation-mediated oxidative stress of soybean exposed to varied sizes of *n*CuO would be size-dependent and that different Cu compounds would induce different biochemical and enzymatic responses in soybean seed. Therefore, recognizing the crucial role of NP size, dose, growth media, and plant species used in nanophytotoxicity (plant growth and development, nutrient quality, and yield) studies, in this study we investigated the potential effects of soil-applied *n*CuO with three different sizes on soybean seed yield attributes and nutrient quality conducting a 120-day full life cycle exposure experiment and propose particle size-dependent seed-Cu bioaccumulation-mediated oxidative stress as a mechanism of action of nCuO toxicity, in soybean. While controlling for surface charge, the tailored synthesis of three distinct particle sizes with high purity enabled investigating particle size-dependent toxicity in soybean, which is a novelty of this work.

## Material and methods

### *nCuO* synthesis, characterization, and localization in seed

Copper oxide nanoparticles (*n*CuO) with three different sizes (small [S] = 25 nm, medium [M] = 50 nm, and large [L] = 250 nm, hereafter denoted as *n*CuO-S, *n*CuO-M, and *n*CuO-L, respectively) were synthesized by sol–gel method. Briefly, copper nitrate trihydrate (Cu(NO_3_)_2_·3H_2_O), citric acid (C_6_H_8_O_7_), and ethylene glycol (C_2_H_6_O_2_) were used in a molar ratio of 1:1:1. The details of the synthesis protocol were reported previously by our group (Yusefi-Tanha et al. 2020b). Phase formation and crystal structure, as well as the particle size distribution of the *n*CuO samples, were characterized using X-ray diffraction (XRD) pattern analysis and field emission-scanning electron microscopy (FE-SEM; FEI Quanta 450 FEG), respectively (see Supplementary Information Fig. S1). Dynamic light scattering (DLS) was used to estimate the hydrodynamic diameter (HDD) and zeta (ζ) potential of the *n*CuO synthesized. Seed embryo ultrastructural changes were imaged with transmission electron microscopy (TEM), and images were analyzed with Digimizer (MedCalc Software Ltd., Belgium).

### Experimental setup

The experiment followed a completely randomized design (RCD). Treatments consisted of control (untreated soil; negative control), CuCl_2_ (Cu^2+^ ions; positive control), and three different *n*CuO sizes (average 25, 50, and 250 nm). CuCl_2_ salt was used as a positive control given its conventional use as a Cu fertilizer and to allow comparison with the published nanophytotoxicity literature (Shi et al. 2011; Apodaca et al. 2017; Ochoa et al. 2017). Each treatment consisted of three pots with each pot containing two plants (*n* = 6 plants per treatment; total 30 plants). The experiment was carried out at Shahrekord University (50° 49′ E, 32° 21′ N), Iran.

### Soil characterization and Cu compounds amendment

The soil was collected at a depth of 0–30 cm, air-dried for 7 days, and sieved (2 mm). The total background Cu concentration in the soil was 0.538 mg/kg. The main physicochemical characteristics of this soil are as follows: classified as silt loam soil (16% sand, 58% silt, and 26% clay), pH = 7.44; EC = 0.47 mmhos/cm; 9.24 g/kg organic matter; 0.88 g/kg total N; 0.011 g/kg available P; and 0.405 g/kg available K. Before planting, 86 kg/ha urea and 100 kg/ha triple superphosphate were added to the culture medium according to the soil test. For soil amendment, different Cu compounds (CuCl_2_; *n*CuO: 25 nm, 50 nm, and 250 nm) were weighed and suspended in 100 mL of distilled water to achieve the desired concentration of 500 mg Cu/kg-soil. The concentration of 500 mg/kg represents various soils with high level of Cu (495–2000 mg/kg-soil) (Ure and Berrow 1982; Holmgren et al. 1993; Niu et al. 2013), which can inhibit plant productivity due to higher Cu bioaccumulation in plant tissues, and this applies to both ionic- and nano-Cu (Rawat et al. 2017; Deng et al. 2020). *n*CuO and Cu^2+^ ions solutions were ultrasonicated (100 W, 40 kHz) for 30 min at 25 °C before mixing with soil using a hand-mixer. After 24 h of equilibration, seeds were sowed in the soil.

### Planting and crop management

This study was conducted in outdoor microcosm conditions to understand the potential phytotoxicity of nanoparticles in the natural field environment. Each polyethylene (PE) pot (20 cm diameter and 20 cm depth) contained 4 kg of soil in a PE bag. To ensure proper drainage, each pot was equipped with an inner PE mesh liner containing 50 holes measuring 5 mm in diameter. Additionally, the bottom of the pot was filled with 500 g of washed gravel to enhance aeration and drainage. Furthermore, to prevent the leaching of Cu and nutrients into the environment, the entire pot was enclosed in a PE bag. The design of the inner PE mesh liner allowed the root system to remain within the pot, facilitating the removal of plants during harvest. For this study, seeds of *Glycine max* cv. Kowsar were obtained from the Seed and Plant Improvement Institute in Iran. Prior to sowing, seeds were imbibed in water for 24 h. Two seeds inoculated with a bacterium, *Rhizobium japonicum*, were planted at a 2.5 cm depth of soil. During the growth period, irrigation was provided at 70% field capacity. During each irrigation event, a sub-sample of water was collected and analyzed using inductively coupled plasma-optical emission spectroscopy (ICP-OES; Varian Vista-Pro Axial) to determine the total Cu concentration. Results showed that Cu concentration in irrigated water was extremely low, ranging from 4 to 5 µg Cu/L, in comparison to the total Cu content in the soil, which was 17 mg Cu/kg soil. Upon reaching maturity, i.e., 120 days post-planting, the plants and seeds were harvested. The seeds were air-dried and stored.

### Copper bioaccumulation in root and seed

For the quantitation of total Cu bioaccumulation in root and seed, the respective samples (0.3 g) were washed several times with Milli-Q water and dried at 70 °C for 48 h. Samples were digested with 10 mL HNO_3_ (150 °C for 1 h), then with 2 mL HClO_4_ at 215 °C for 2 h (5:1 v/v). The digests were diluted to 10 mL using deionized water. The extracts were filtered prior to ICP-OES analysis for total Cu concentrations (Ghasemi Siani et al. 2017). Six-point calibration curves were developed, and the detection limit was 30 µg Cu/L. Blank constituted Milli-Q water with 2% HNO_3_.

### Measurement of photosynthetic pigments

For the measurement of photosynthetic pigments, one of the youngest leaves per plant (two leaves per pot) were sampled, at the flowering stage. Then, 100 mg of fresh leaf tissue were weighed and ground with 5 mL of 80% acetone using a ceramic mortar and pestle until a homogeneous mixture is attained. After filtering of resulting extract with funnel and filter paper, the obtained extract was made up to 10 mL with 80% acetone. The absorbance of the extract was read at 663.2, 646.8, and 470 nm wavelengths, and the chlorophylls (chla and chlb) and carotenoids values were calculated following Lichtenthaler and Buschman (2001). The values are reported in mg/g of plant tissue fresh weight (FW).

1 $${\text{Chla}}\;\left({\text{mg}}/{\text{mL}}\right)=12.5\times {A}_{663.2}-2.79\times {A}_{646.8}$$

2 $${\text{Chla}}\;\left({\text{mg}}/{\text{mL}}\right)=21.51\times {A}_{646.8}-5.1\times {A}_{663.2}$$

3 $$\mathrm{Carotenoids}\;(\text{mg}/\text{mL})=\left[1000\;\left(A_{470}\right)-1.82\;\left(\text{Chla}\right)-85.02\;\left(\text{Chlb}\right)\right]/198$$

where *A* is the light absorbed by the extract at corresponding wavelengths (shown as subscripts).

### Measurement of yield attributes

Plants, pods, and seeds were harvested 120 days after sowing, when the pods turned brown (Kamali-Andani et al. 2023). After counting the number of pods/plant and the number of seeds/pod, the seeds were air-dried and weighed using a digital weighing balance. To determine the amount of seed phosphorus and potassium, after seed drying, grinding, and sieving, the samples were digested and measured using a spectrophotometer (Khoshgoftarmanesh 2007) and a flame photometer (Watson and Isaac 1990), respectively. The seed oil was extracted using the standard Soxhlet extraction method. Seeds (15 g) were weighed and powdered. Then, the sample was poured into the extraction thimbles, and hexane solvent (about 300 mL) was poured into the device balloon. After heating the balloon for 4 h when the solvent color changed, the mixture of solvent + sample oil was transferred to a rotary device. The oil content (%) was calculated following Eq. (4) (Assadi et al. 2014):

4 $${\text{Oil}}\;(\mathrm{\%})=({\text{SSW}}/{\text{OW}})\times 100$$

where SSW and OW denote seed sample weight (g) and oil weight (g), respectively.

The seed samples were digested after drying, grinding, and sieving. Then, the nitrogen concentration of seed was measured by the Kjeldahl method (Bremner 1996), and seed protein was calculated following Eq. (5) (Olama et al. 2013):

5 $$\mathrm{Protein }\;\left(\mathrm{\%}\right)={\text{SN}}\times 6.25$$

where SN denotes seed nitrogen (%).

Oil and protein yields were calculated as follows (Ghanbari et al. 2019):

6 $$\mathrm{Oil\; yield }\;({\text{g}}/{\text{plant}})={\text{SY}}\times {\text{SO}}$$

7 $$\mathrm{Protein\; yield }\;({\text{g}}/{\text{plant}})={\text{SY}}\times {\text{SP}}$$

where SY, SO, and SP denote seed yield (g), seed oil (%), and seed protein (%), respectively.

### Measurement of oxidative stress biomarkers

Two youngest leaves per pot were sampled to determine a suite of oxidative stress biomarkers: hydrogen peroxide (H_2_O_2_), malondialdehyde (MDA), superoxidase dismutase (SOD), catalase (CAT), superoxidase dismutase (SOD), guaiacol peroxidase (POX), and ascorbate peroxidase (APX). The details of antioxidative enzymes measurement were reported previously by our group (Yusefi-Tanha et al. 2020a, b), and briefly described below.

Lipid peroxidation was determined in leaf by measuring the formation of MDA content with thiobarbituric acid (TBA) using the method of Heath and Packer (Heath and Packer 1968). Briefly, fresh leaf samples (0.1 g) were homogenized in 1.5 mL of 0.1% trichloroacetic acid (TCA). The resultant homogenate was centrifuged at 10,000 × *g* for 10 min, and 1 mL of the supernatant was added to 2 mL of 20% TCA containing 0.5% TBA. The extract was heated in water bath (95 °C, 30 min), then cooled in ice bath before centrifugation (10,000 × *g*, 10 min). The absorbance of the supernatant was measured at 532 nm and 600 nm and blank corrected. The MDA content was expressed as nmol g^−1^ FW (using the extinction coefficient of 155 mM^−1^ cm^−1^) (Narwal et al. 2009).

The H_2_O_2_ levels were measured following Nag et al. (2000). Briefly, fresh leaf tissue (1 g) was powdered using liquid nitrogen and was homogenized in 12 mL cold acetone. Then, homogenate was filtered through the Whatman filter paper. The mixture was diluted using 4 mL titanium (16%), and 0.2 mL ammonium hydroxide (28%). The tissue extract was further centrifuged at 8500 rpm for 5 min at 4 °C. The supernatant was isolated, then the precipitate washed twice with 5 mL of acetone. Two mL of sulfuric acid (1 M) was added to the precipitate and absorption measured at 410 nm. The H_2_O_2_ concentration was expressed as nM g^−1^ FW.

The SOD is a major O_2_^•−^-scavenging enzyme in cytosol, mitochondria, chloroplast, and peroxisome, which converts O_2_^•−^ into H_2_O_2_ (Demidchik 2015). Following the method by Narwal et al. (2009), leaf SOD activity was measured as inhibition of the photochemical reduction of nitroblue tetrazolium (NBT). One unit of SOD activity is defined as the amount of enzyme that causes 50% inhibition of oxidation reactions per mg of protein in extract. One g of fresh leaf sample was frozen in liquid nitrogen, homogenized in 10 mL of 0.1 M potassium phosphate buffer (pH = 7.5), and centrifuged at 20,000 rpm for 30 min at 4 °C. The supernatant was collected, separated into aliquots, and stored at − 20 °C. 1.95 mL of 0.1 M potassium phosphate buffer (pH 7.5), 250 μL of 150 mM methionine, 250 μL of 1.2 mM Na_2_EDTA, 250 μL of 24 μM riboflavin, 250 μL of 840 μM NBT, and 50 μL of plant extract were prepared. The reaction was initiated by light illumination, and the absorbance was read at 560 nm.

The CAT is a key enzyme that breaks H_2_O_2_ molecules into H_2_O and O_2_, and maintains an optimum level of H_2_O_2_ for cellular signaling processes (Nandi et al. 2019). Following the method by Narwal et al. (2009), leaf CAT activity was determined by measuring the decrement in absorbance at 240 nm following the decomposition of H_2_O_2_. One unit of CAT activity corresponds to 1 mM of H_2_O_2_ consumed per min per mg of protein using an extinction coefficient of 40 mM^−1^ cm^−1^. Briefly, the reaction mixture consisted of 50 μL of supernatant, 1.95 mL of 0.1 M potassium phosphate buffer (pH 7.0), and 100 μL of 264 mM H_2_O_2_. The decrease in absorption was recorded for 100 s at 5-s intervals at room temperature (25 °C).

The POX works in the extracellular space for scavenging H_2_O_2_ and prevents the formation of more harmful ROS by H_2_O_2_ (Rajput et al. 2021). Following the protocol by MacAdam et al. (1992), we estimated leaf POX activity. One unit of POX activity corresponds to 1 mM tetraguaiacol consumed per min per mg of protein using an extinction coefficient of 26.6 mM^−1^ cm^−1^. Briefly, 50 μL of plant extract was added to 1.35 mL 0.1 M potassium phosphate buffer (pH 6.0), 100 μL 45 mM guaiacol, and 500 μL 44 mM H_2_O_2_. Then, we measured changes in absorbance at 470 nm at 10-s intervals for 300 s at 25 °C using an UV–Vis spectrophotometer.

The APX reduces H_2_O_2_ to H_2_O and mono-dehydroascorbic acid (MDHA), using ascorbic acid as a reducing agent, particularly in the cytosol and chloroplast (Ding et al. 2022). One unit of APX is defined as 1 mM of ascorbate oxidized per min per mg of protein, and the method followed Narwal et al. (2009). APX activity measures the rate of ascorbate oxidation with H_2_O_2_, following the method developed by Narwal et al. (2009). The decrease in ascorbic acid, starting from a mixture of 2.4 mL of 0.1 M potassium phosphate buffer (pH 7.0), 250 μL of 1.2 mM Na_2_EDTA, 50 μL of 35 mM H_2_O_2_, 100 μL of 15 mM ascorbic acid, and 200 μL of supernatant was measured at 290 nm over 500 s at 10-s interval at room temperature (25 °C). The activity was calculated using an extinction coefficient of 2.8 mM^−1^ cm^−1^.

### Statistical analysis

A one-way analysis of variance (ANOVA) was performed using SAS (SAS Inc., ver. 9.4) to examine significant differences in crop responses to different Cu compounds following a completely randomized experimental design (CRD). A Fisher LSD test at the 0.05 probability level was used to compare the means between treatments. The results are presented as mean ± standard deviation (SD).

## Results and discussion

### Nanoparticle characterization

The XRD analysis revealed that the *n*CuO samples were monoclinic crystalline without any noticeable trace of impurities (Fig. S1, left panels). FE-SEM micrographs showed mean particle size of 25 nm, 50 nm, and 250 nm, for *n*CuO-S, *n*CuO-M, and *n*CuO-L, respectively (Fig. S1, right panels), and DLS analysis showed that their HDDs were 189.0 nm, 195.1 nm, and 915.6 nm, respectively. The average zeta potential for these three distinct sized *n*CuO were similar in the range (− 51.5)–(− 52.6) mV, thus allowing for elucidating potential particle size-dependent effects. Additional details on *n*CuO characterization data were previously reported in our companion papers (Yusefi-Tanha et al. 2020a, b).

### Cu bioaccumulation in root and seed

**Fig. 1:**
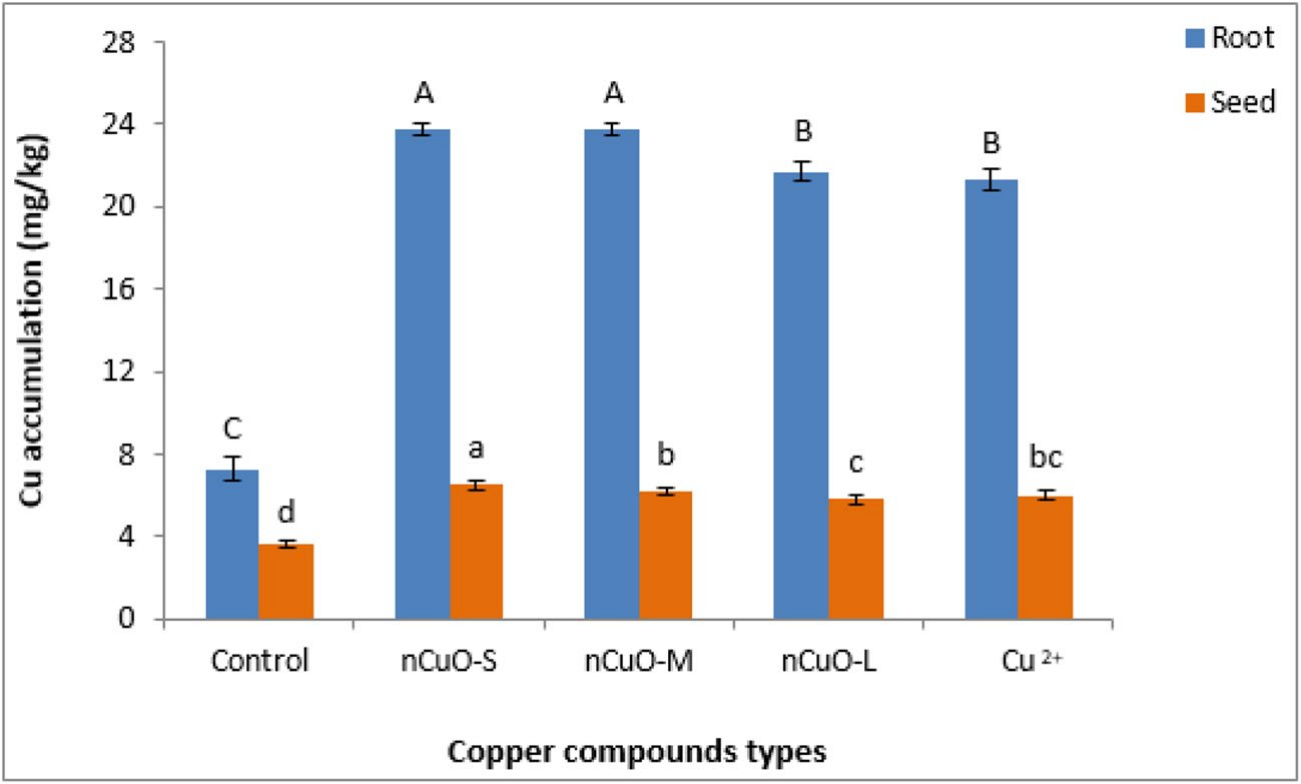
Cu accumulation in soybean root and seed upon exposure to soil-amended *n*CuO-25 nm, *n*CuO-50 nm, *n*CuO-250 nm, and CuCl_2_, at 500 mg/kg-soil. Bars represent mean ± SD. Different letters above the bar indicate significant difference at *p* < 0.05 according to the LSD test

Results showed that the effects of Cu compound type (Cu_type_) were significant for Cu bioaccumulation in root (*p* < 0.0001) and seed (*p* < 0.0001) and were particle size-dependent (Table S1). For all Cu compound types, Cu bioaccumulation in root significantly increased by over threefold compared to untreated control, with *n*CuO-25 nm and *n*CuO-50 nm having the highest root Cu bioaccumulation (Fig. 1). Furthermore, Cu bioaccumulation in root was significantly greater with *n*CuO-25 nm treatment compared to the larger size *n*CuO-250 nm or Cu^2+^ ions treatments. Between Cu^2+^ ions and *n*CuO-250 nm, Cu bioaccumulation in root were not statistically significant (*p* > 0.05). The Cu bioaccumulation in seed exhibited a pattern similar to root, with the seed showing approximately 3.5 times lower Cu bioaccumulation compared to the root. This difference was particularly evident in the nCuO-25 nm treatment (Fig. 1). Our findings are consistent with the results previously reported by Ogunkunle et al. (2018).

It can, thus, be surmised that the root being in direct contact with the soil tends to sorb a higher amount of Cu while the seed that is developed later in life and farthest away from the root seems to bioaccumulate the lowest amount of Cu. Higher Cu bioaccumulation in root and seed with *n*CuO-25-nm treatment might reflect smaller size-facilitated NP transport across the cellular barriers, considering that plasmodesmata or intercellular bridges are around 40 nm in diameter, just big enough for 25-nm particles to traverse through (Tilney et al. 1991; Dietz and Herth 2011; Andreotti et al. 2015).

### Photosynthesis apparatus

**Fig. 2:**
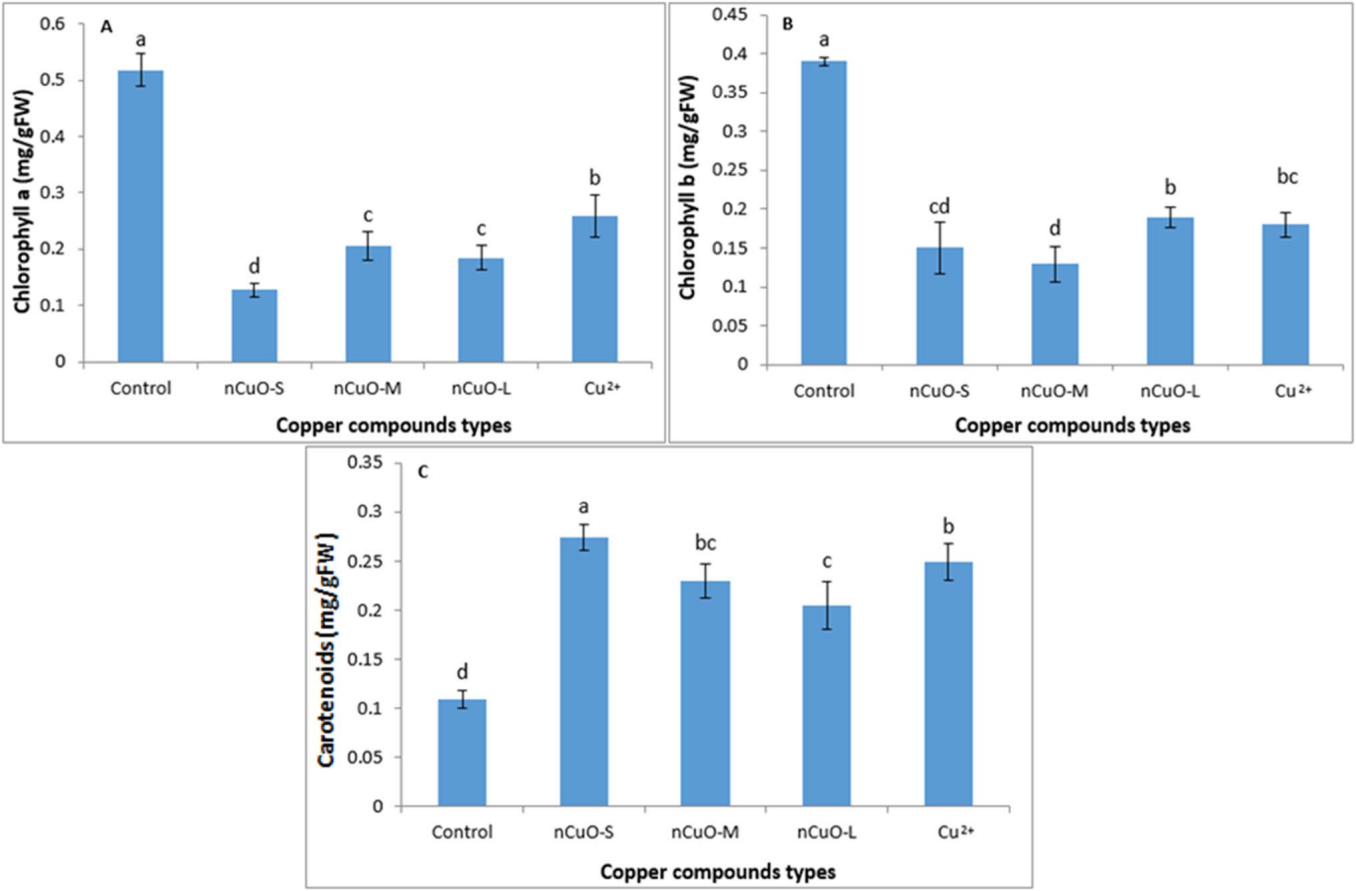
Effect of nano copper oxide (*n*CuO) and copper chloride (CuCl_2_) on chlorophyll-*a* (**A**), chlorophyll-*b* (**B**), and carotenoids (**C**). *n*CuO-S, *n*CuO-M, and *n*CuO-L represent *n*CuO-25 nm, *n*CuO-50 nm, and *n*CuO-250 nm, respectively, at 500 mg/kg-soil. Bars represent mean ± SD. Different letters above the bar indicate significant difference at *p* < 0.05 according to the LSD test

Based on ANOVA, the Cu compound types (*n*CuO and Cu^2+^) significantly affected the photosynthetic pigments (Chla, Chlb, and carotenoids) in soybean (Table S2). The results showed that, generally, all Cu compounds significantly reduced the levels of Chla and Chlb in soybean, compared to untreated control (*p* < 0.05) (Fig. 2A, B), and that the reduction was significantly higher with the smaller-sized *n*CuO. In plants treated with *n*CuO-S, the levels of Chla and Chlb were 75 and 61.5% lower than control, respectively. Interestingly, the toxicity trend was reversed for carotenoids, whereby *n*CuO-S significantly promoted carotenoids compared to larger-sized *n*CuO, Cu^2+^ ions, and control (Fig. 2C). On average, the carotenoids content was 2.5-fold higher with *n*CuO-S treatment compared to control. With *n*CuO-M treatment, the level of carotenoids did not differ significantly compared to *n*CuO-L and Cu^2+^ ions treatments.

Copper is an essential component of various proteins such as plastocyanin of the photosynthetic apparatus, and cytochrome oxidase of the respiratory electron transport chain (Asati et al. 2016). A decrease in chlorophyll may manifest in the reduction of leaf thickness and anatomy or may be a result of limited bioavailability of mineral nutrients, such as Mn^2+^, Zn^2+^, Fe^2+^, and Mg^2+^ owing to antagonistic effect of Cu on mineral bioavailability (Lequeux et al. 2010; Feigl et al. 2013). Fe^2+^ and Mg^2+^ deficiency is known to inhibit chlorophyll biosynthesis, leading to reduced photosynthesis (Küpper and Kroneck 2005). Furthermore, excess Cu leads to the increase in superoxide radicals and single oxygen in chloroplast through the Fenton reaction. These radicals attack compounds with double bonds such as chlorophyll, causing the release of chlorophyll from the thylakoid membrane and reducing its content (Zhang et al. 2003).

Chla, the major photosynthetic pigment in plants, contains a methyl (-CH_3_) group whose key function is to bind a photon and is more sensitive to photodegradation than other pigments (Barker and Pilbeam 2015; Rico et al. 2015). Chlb plays an important role in improving light absorption efficiency and thus increasing energy production and biomass in plants (Xiao et al. 2022). The Chlb content was significantly affected by 500 mg/kg *n*CuO (Xiao et al. 2022), which is consistent with our results. In *Brassica juncea* L., *n*CuO also reduced chlorophyll and carotenoids levels (Nair and Chung 2015), while in *Coriandrum sativum*, Cu-based NPs did not affect chlorophyll production (Zuverza-Mena et al. 2015). Da Costa and Sharma (2016) reported that the accumulation of *n*CuO in *Oryza sativa* chloroplasts reduced the number of thylakoids per grana, photosynthetic pigment synthesis, rate of photosynthesis, transpiration, stomatal conductance, and quantum efficiency. Carotenoids are auxiliary pigments that, in addition to absorbing light by preventing the formation of reactive oxygen species, protect the photosynthetic apparatus against the damage of additional photons and oxidative stress (by the xanthophyll cycle) (Shaw and Hossain 2013). Our results showing an inverse relationship of carotenoids with *n*CuO sizes (Fig. 2C) indicate the antioxidative role of carotenoids. Previously, the amount of Chla and Chlb in *Landoltia punctata* decreased with *n*CuO (70 nm) treatment, while the carotenoid levels increased compared to control, which is consistent with our results (Fig. 2) (Lalau et al. 2015). In a study conducted by Gopalakrishnan Nair et al. (2014), total chlorophyll content was significantly reduced at 500 mgL^−1^ of *n*CuO (25–50 nm) compared to control. However, carotenoid content did not change significantly.

### Pod formation and seed yield

**Fig. 3:**
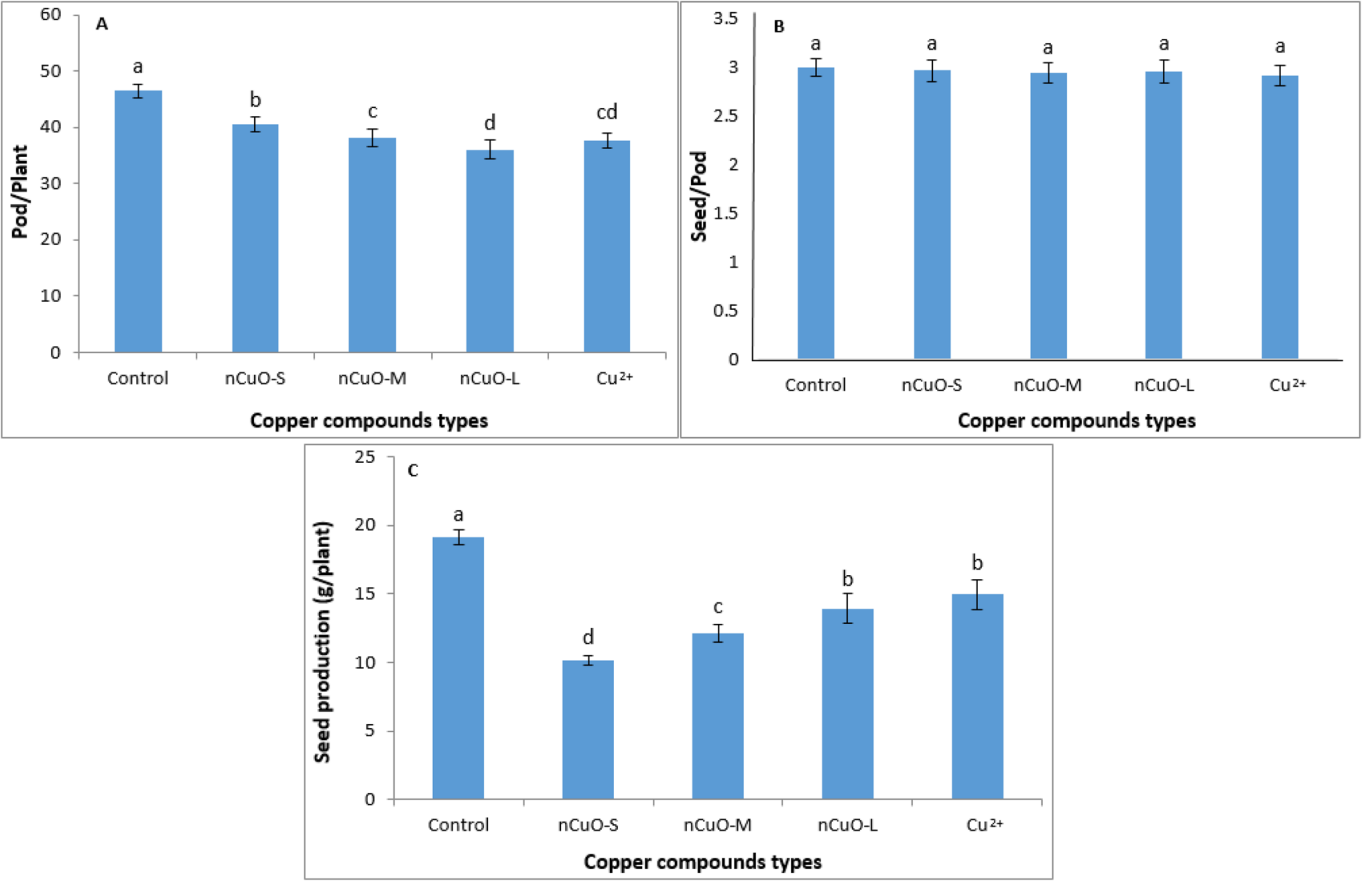
Effect of copper compound types on pod number per plant (**A**), seed number per pod (**B**), and seed production (**C**) in soybean. *n*CuO-S, *n*CuO-M, and *n*CuO-L represent *n*CuO-25 nm, *n*CuO-50 nm, and *n*CuO-250 nm, respectively, at 500 mg/kg-soil. Bars represent mean ± SD. Different letters above the bar indicate significant difference at *p* < 0.05 according to the LSD test

Based on ANOVA, the Cu compound types (*n*CuO and Cu^2+^) significantly affected pod formation and seed yield (*p* < 0.01) but had no impact on seed formation (*p* > 0.05), in soybean (Table S2). The pod formation and seed yield under the influence of different-sized *n*CuO and Cu^2+^ are shown in Fig. 3. Increasing the size of *n*CuO decreased the pod number per plant compared to control. Larger-sized *n*CuO (M and L) and Cu^2+^ ions treatments had no significant difference in pod number per plant (Fig. 3A). Further, seed per pod was not affected by different sizes of *n*CuO and Cu^2+^ compared to control (Fig. 3B). However, seed production (g/plant) was particle size-dependent, with smaller size inhibiting seed production significantly. For *n*CuO-S treatment, seed production was inhibited by 48% compared to control (Fig. 3C).

Soybean pods contain a number of seeds and provide photosynthetic assimilates needed for seed development, which determines seed weight (i.e., seed production) (Monica and Cremonini 2009; Seyed Sharifi and Khoramdel 2016; Wijewardana et al. 2019). In the present study, although the pod number was higher and the seed number per pod was unaffected (Fig. 3A, B), seed weight/production per plant treated with *n*CuO was lower than Cu^2+^ ions treatment (Fig. 3C). These results suggest that due to the decrease in the Chla and Chlb synthesis (Fig. 2A, B) with *n*CuO-S treatment, plants likely were unable to provide photosynthetic materials during seed filling, leading to decreased seed weight/production (Fig. 1C).

### Seed nutrient quality

#### Phosphorus (P) and potassium (K)

**Fig. 4:**
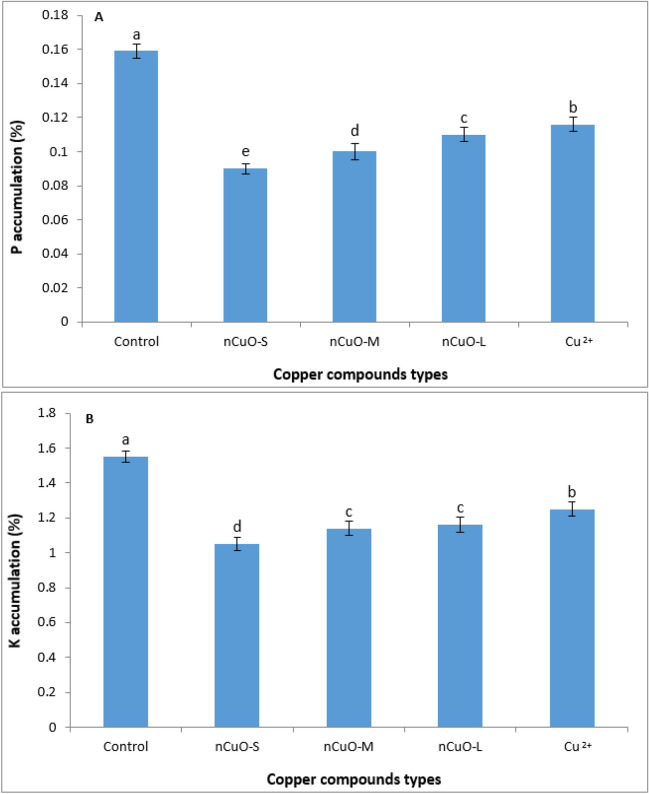
Effect of nano copper oxide (*n*CuO) and copper chloride (CuCl_2_) on phosphorus (**A**) and potassium (**B**) accumulation in soybean seed. *n*CuO-S, *n*CuO-M, and *n*CuO-L represent *n*CuO-25 nm, *n*CuO-50 nm, and *n*CuO-250 nm, respectively, at 500 mg/kg-soil. Bars represent mean ± SD. Different letters above the bar indicate significant difference at *p* < 0.05 according to the LSD test

Our results showed that the seed quality (P, K, protein, and oil) was affected by the Cu compound types (*p* < 0.01, Table S3). The P and K content in soybean seed is shown in Fig. 4. P content was significantly reduced with the decrease in *n*CuO size. Compared to control, a 43% and 32% reduction in P and K content, respectively, were observed in soybean seed when exposed to *n*CuO-S, while a 30.8% and 25% reduction in P and K content, respectively, were observed for *n*CuO-L (Fig. 4A, B). Consistent with our findings, a previous study documented altered nutritional quality (higher Cu, S, and Al, but lower Mg, Ca, P, and Mn) in lettuce treated with Cu-based NPs compared to control (Trujillo-Reyes et al. 2014). Likewise, a recent study reported a decrement in K, Mg, Zn, and Ca levels by up to 47.4%, 34.3%, 37.6%, and 60.0%, respectively, with 75 and 150 mg/kg nCuO treatments in weedy rice grains, but no such decreases were noted in cultivated rice, and Fe levels increased by up to 88.6%, and 53.2%, with 75 and 150 mg/kg nCuO treatments, respectively (Deng et al. 2022).

The *n*CuO may have diverse mechanisms of toxicity due to their special properties, including specific surface area and high surface energy (Rawat et al. 2017). Soil-applied NPs may compete with nutrient elements in the soil, potentially disrupting nutrient uptake (Thounaojam et al. 2012; Peralta-Videa et al. 2014). Cu ion dissolution from *n*CuO within the soil-root interface can form complexes with phosphate ions (H_2_PO_4_^−^ and HPO_4_^2−^), limiting P bioavailability (Rawat et al. 2017). Further, physical blocking of membrane transporters by *n*CuO may also lead to reduced P uptake (Zuverza-Menaet al. 2015). In a previous study, P concentration in *Capsicum annum* L. fruits treated with 500 mg/kg *n*CuO was significantly lower compared to Cu^2+^ ions treatment (Rawat et al. 2017). *n*CuO also had a significant inhibitory effect on P transfer to leaf and fruit, likely due to aggregation of the NPs and the relatively better diffusion of Cu^2+^ ions in the soil. Because aggregation of NPs reduces their surface area and dissolution potential, especially with larger sizes (Baker et al. 2014), this may have led to the decrease in seed P with the larger-sized *n*CuO (M and L) compared to *n*CuO-S in our study (Fig. 4A). Consistent with our results, a reduction in root, leaf, and fruit P in *Medicago sativa* and *Lactuca sativa* exposed to *n*CuO was reported (Hong et al. 2015). It is known that P uptake by root from soil is controlled by specialized transporters, while movement within plant tissues is due to other transporters. Phosphate transporters 1 (Pht1) are specific for obtaining P from the soil, while phosphate transporters 2 (Pht2) are responsible for the transport of P from root to leaf and fruit through the stem (Buchner et al. 2004; Hong et al. 2015). Future studies should explore the putative role of such transporters in P transport under *n*CuO stress.

Like P, K is another essential nutrient with role in many biochemical and physiological processes in plants, including the transport of water and nutrients. High concentration of K can improve fruit physical quality and nutritional value (Servin et al. 2013). Our results showed that all Cu compounds decreased seed K compared to control (Fig. 4B), which can affect the seed quality and nutritional value of soybean. Decreased K levels may indicate membrane leakage in plants exposed to *n*CuO. Wang et al. (2012) showed that *n*Cu increased K leakage in *Zea mays* L. root and shoot. Further, the combined effect of negative surface charge and higher surface-to-volume ratio of *n*CuO may promote complex formation with K^+^ and reduce its bioavailability (Deng et al. 2022). Our results are consistent with previous studies conducted in *Phaseolus vulgaris* and *Brassica rapa*. Different Cu compounds negatively affected K uptake and accumulation in *P. vulgaris* shoots and leaves (Apodaca et al. 2017). In *B. rapa*, leaf K was significantly reduced (45%) with 150 mg/kg *n*CuO compared to control (Deng et al. 2020). A positive interaction of K with N and P has also been reported (Barker and Pilbeam 2015). Optimal nutrient balance is crucial to maximize yield and quality, and metal toxicity may manifest via a disturbance in the nutritional balance, resulting in the deficiency of essential nutrients and impacting seed quality and nutritional value (Barker and Pilbeam 2015).

#### Seed protein

**Fig. 5:**
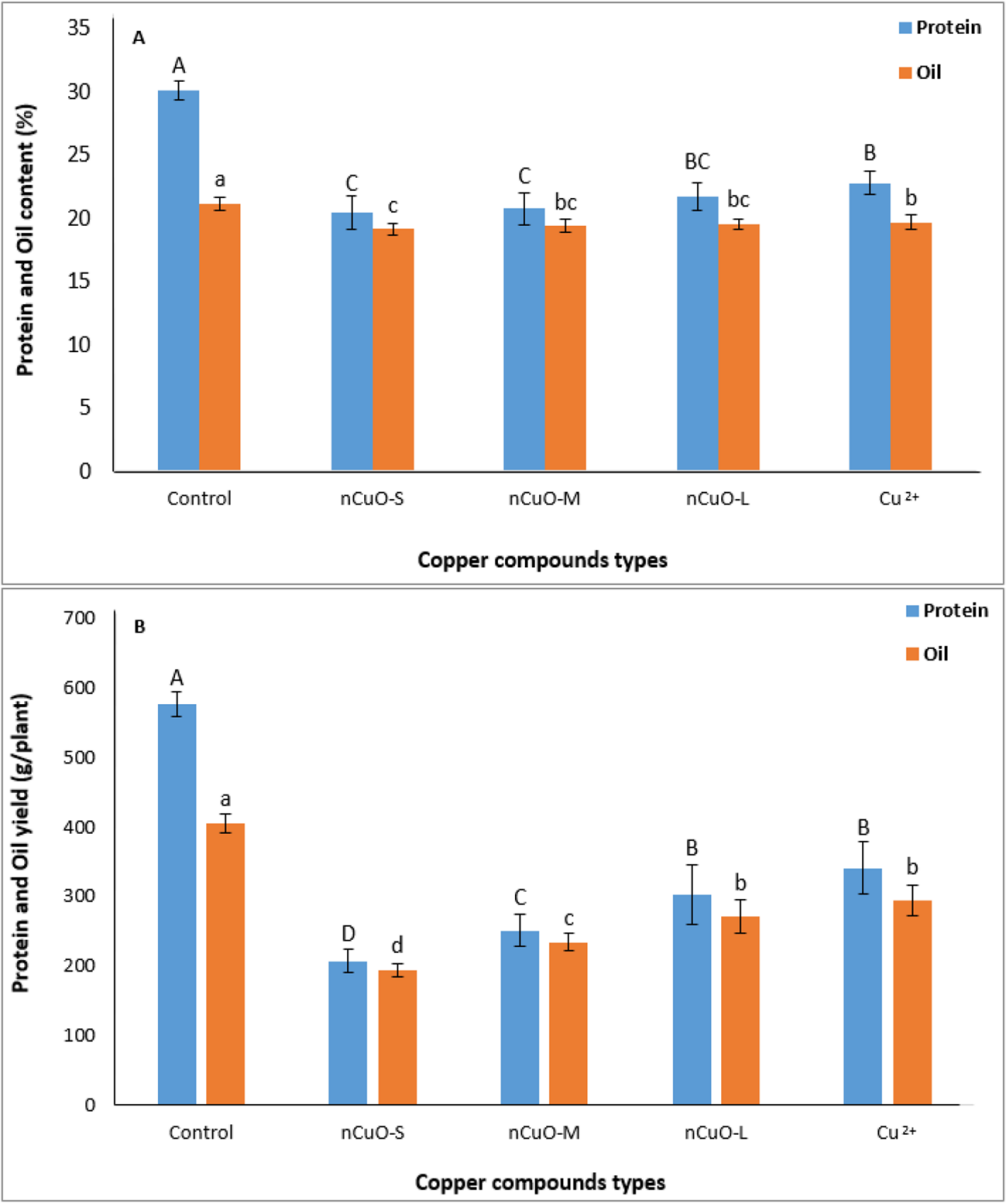
Effect of copper compound types on protein and oil content (**A**) and protein and oil yield (**B**) in soybean seed. *n*CuO-S, *n*CuO-M, and *n*CuO-L represent *n*CuO-25 nm, *n*CuO-50 nm, and *n*CuO-250 nm, respectively, at 500 mg/kg-soil. Bars represent mean ± SD. Different letters above the bar indicate significant difference at *p* < 0.05 according to the LSD test

As depicted in Fig. 5, all Cu compound types significantly decreased the seed protein. Overall, *n*CuO-S showed the lowest protein content, which was on average 32% lower than control, but this was not significantly different among the different *n*CuO sizes (Fig. 5A). Likewise, the *n*CuO-S, M, and L decreased seed protein yield from 576 g/plant (for control) to 207, 251, and 303 g/plant, respectively (Fig. 5B). In addition, protein yield in plants treated with nCuO-L and Cu^2+^ did not differ significantly (Fig. 5B).

Soybean seed protein content is determined by N uptake capacity and synthesis of proteins stored in the growing seed (Wang et al. 2019). In soybean, the amount of total essential amino acids is positively correlated with seed protein (Zhang et al. 2018). Therefore, the reduction of the amount of seed protein under the influence of *n*CuO can be related to the direct effect on N needed for the biosynthesis of protein constituents including amino acids. Both N and ammonium are needed for the synthesis of amino acids to ultimately form proteins. Amino acids are also used for the synthesis of chlorophyll (Barker and Pilbeam 2015). Reduction in N and subsequent reduction in chlorophyll (Fig. 2A, B) could decrease photosynthesis and thus plant productivity. Whereas optimal Cu promotes photosynthesis and chloroplast protein (Rai et al. 2018), its excess can impair net photosynthesis vis-a-vis seed protein levels.

#### Seed oil

Generally, from the perspective of changes in seed quality, with the decrease in *n*CuO size, a decrease in seed oil was observed (Fig. 5). Soybean exposed to *n*CuO-S showed the lowest oil content, which was 9.5% lower compared to control, but it was not significantly different from larger-sized *n*CuO (M and L). Also, no significant difference was observed in oil content between larger-sized *n*CuO and Cu^2+^ (Fig. 5A). With the *n*CuO exposure, the different sizes (25, 50, and 250 nm) significantly reduced oil yield by 52, 42, and 32.7%, respectively, when compared to control. The oil yield in plants treated with *n*CuO-L and Cu^2+^ ions showed no significant difference (Fig. 5B).

A reduction in photosynthesis with *n*CuO treatments could impair C allocation for protein and oil synthesis (Hernandez-Sebastia et al. 2005). Further, the amount of protein depends more on C and N remobilization from leaves, while the amount of oil depends more on the current photosynthesis (Wang et al. 2019). In our study, inhibition of photosynthetic pigments, Chla and Chlb (Fig. 2A, B), and reduced bioavailability of micronutrients such as P and K (Fig. 4), may have a direct bearing in decreased protein and oil content and yield in soybean seed.

### Seed ultrastructure

**Fig. 6:**
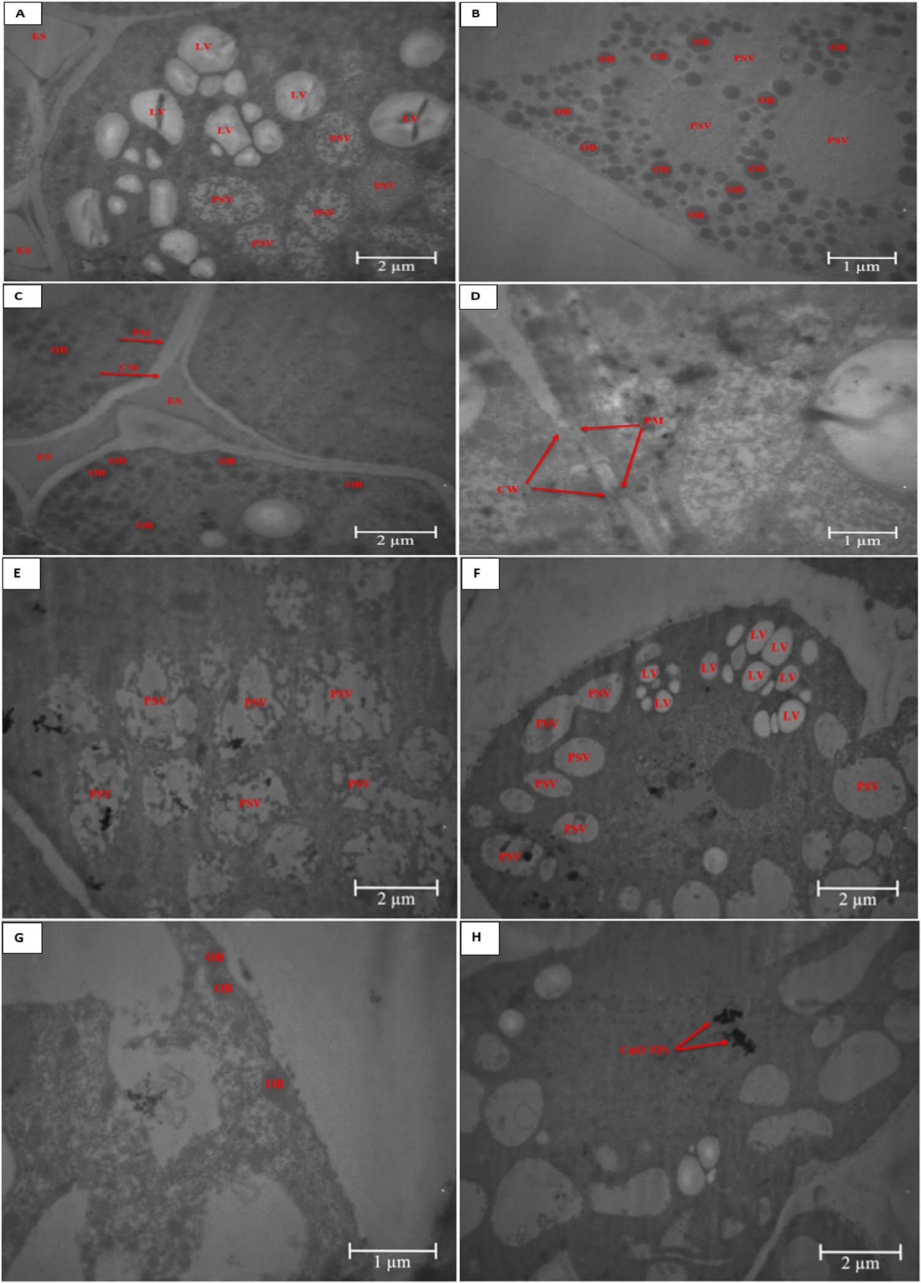
TEM analysis of soybean seed embryo ultrastructure upon treatment with *n*CuO-S at 500 mg/kg soil (**D**–**H**) and untreated control (**A**–**C**). PSV, protein storage vacuoles; OB, oil bodies; LV, lytic vacuoles; CW, cell wall; PM, plasma membrane; ES, extracellular space

TEM imaging of soybean seed embryo ultrastructure showed that most of the seed embryo cytoplasm contained storage proteins. Oil bodies containing seed oil fill the spaces between the storage proteins, and both (storage proteins and oil bodies) are embedded in the cytoplasmic network of the cell (Fig. 6A, B). TEM images showed normal morphology of cell wall and plasma membrane of untreated soybean seed embryo cells (Fig. 6C). On the other hand, the integrity of cell wall and plasma membrane appeared to be perturbed in plants treated with *n*CuO-S (Fig. 6D). Mirzajani et al. (2013) reported that NPs at a high concentration can cause damage to the cell wall and plasma membrane, enabling them to enter and disturb different functions in plant. The number of protein storage vacuoles in plants treated with *n*CuO-S did not differ from control, but their size (on average 1.39 µm) increased compared to control (on average 1.29 µm). The treatment of nCuO-S resulted in modification of protein storage vacuoles’ shape and a disruption of their structural integrity (Fig. 6E, F). This alteration potentially contributed to a reduction in protein content compared to untreated control (Fig. 5A). The number of oil bodies in seed embryo of plants treated with *n*CuO-S decreased (Fig. 6B, G), leading to a reduction in oil content (%) compared to control (Fig. 5A). Nanoparticles can cross the cell membrane and form agglomerates with themselves or other intracellular substances. In the current study, it is speculated that *n*CuO may have passed through the cell membrane and agglomerated in the cytoplasm of embryo (Fig. 6H). The putative accumulation of NPs in the seed of soybean merits further investigation into potential health risk to consumers, including humans.

### Biomarkers of oxidative stress response

**Fig. 7:**
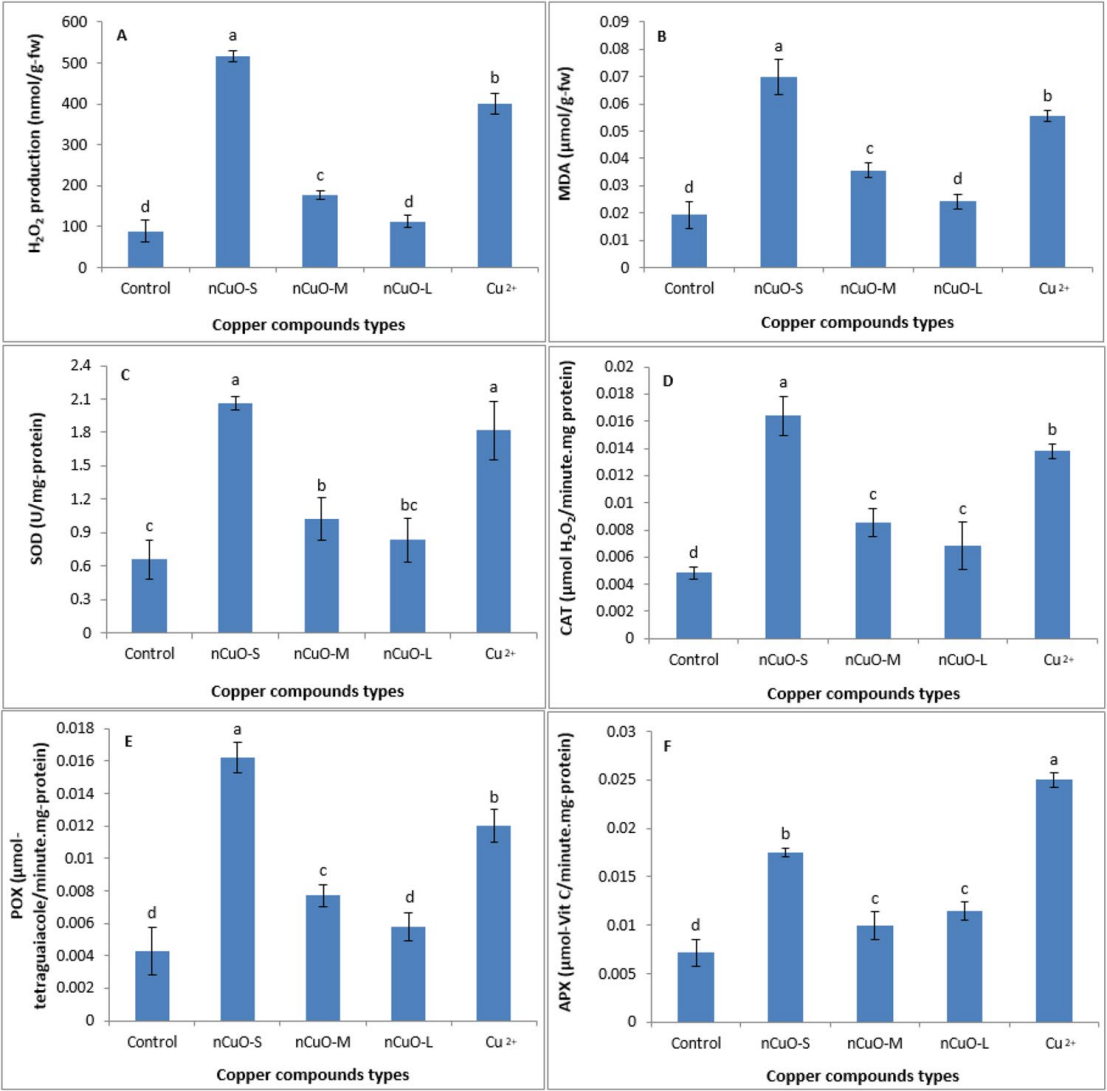
Changes in leaf hydrogen peroxide (H_2_O_2_) production (**A**), malondialdehyde (MDA) content (**B**), superoxide dismutase (SOD) (**C**), catalase (CAT) (**D**), guaiacol peroxidase (POX) (**E**), and ascorbate peroxidase (APX) (**F**) in soil grown soybean treated with *n*CuO-25 nm, *n*CuO-50 nm, *n*CuO-250 nm, and CuCl_2_, at 500 mg/kg-soil. Bars represent mean ± SD. Different letters above the bar indicate significant difference at *p* < 0.05 according to the LSD test

Oxidative stress has predominantly been documented as a toxicity mechanism underlying nanomaterial exposure in various organisms (Tee et al. 2016). In this study, a suite of oxidative stress biomarkers was evaluated as a response to *n*CuO exposure in soybean grown for a full life cycle of 120 days. The Cu compound types significantly affected the antioxidative enzymes, H_2_O_2_, and MDA in soybean (Tables S1, S4). Results showed significantly elevated levels of H_2_O_2_ and MDA in soybean leaf upon *n*CuO treatments compared to the untreated control, and the effects were particle size dependent with *n*CuO-25-nm treatment showing the highest H_2_O_2_ and MDA concentrations while *n*CuO-250 nm had the lowest H_2_O_2_ and MDA concentrations (Fig. 7A, B). To counteract the oxidative stress elicited by stressors, including nanomaterials, plants are known to synthesize a gamut of antioxidative enzymes, including SOD, CAT, POX, and APX, among others (Dogaroglu and Koleli 2017; Ogunkunle et al. 2018). In this study, we measured the leaf concentrations of antioxidative enzymes: SOD, CAT, POX, and APX, in soybean treated with three distinct sized *n*CuO. Our results showed that, among the NPs used, the tested antioxidant levels were the highest with *n*CuO-25 nm treatment and the lowest with *n*CuO-250 nm treatment. These results suggest a direct response of the plant antioxidant system to counteract the higher oxidative stress incurred by higher H_2_O_2_ and MDA levels upon *n*CuO treatments. Akin to the MDA and H_2_O_2_ synthesis, the antioxidative responses (i.e., SOD, CAT, POX, and APX) were particle size-dependent (Fig. 7C–F). Furthermore, with the *n*CuO-250-nm treatment, the oxidative stress biomarkers (MDA and H_2_O_2_) and antioxidant biomarkers’ (i.e., SOD, CAT, POX, and APX) concentrations were generally similar to control. On the other hand, Cu^2+^ ion treatment showed significantly higher H_2_O_2_ and MDA levels compared to *n*CuO-50-nm and *n*CuO-250-nm treatments, but these biomarker levels were significantly lower than *n*CuO-25-nm treatment. In response, the antioxidant (i.e., SOD, CAT, POX, and APX) levels also increased significantly, mirroring the trends of oxidative stress biomarkers (MDA and H_2_O_2_) (Fig. 7).

It is, however, unclear if the MDA synthesis is directly related with *n*CuO or Cu^2+^ ion impact on the lipid peroxidation of the cell membrane, or indirectly mediated through the release of reactive oxygen species (ROS) such as H_2_O_2_ as measured in this study. Nonetheless, higher H_2_O_2_ synthesis is expected to cause higher accumulation of MDA, a byproduct of membrane lipid peroxidation, as observed in our study for *n*CuO-25 nm and Cu^2+^ ions.

Taken together, the following mechanism of action is proposed: upon root uptake of *n*CuO or Cu^2+^ ions, they were transported to and bioaccumulated in seed, leading to oxidative stress, which proportionally affected photosynthetic pigments, seed yield/production, and seed nutrient quality (i.e., protein, oil, P, and K) as a function of particle size.

Particle size, surface charge, concentration, and type of NPs have consistently been documented to affect their absorption, translocation, and bioaccumulation in plants (Kaphle et al. 2018; Yusefi-Tanha et al. 2020a; Mittal et al. 2020). NPs with sizes smaller than that of the pores in cell wall have greater transport potential (Dietz and Herth 2011), while larger NPs may face difficulty traversing through (Iram et al. 2023). With soil application, NPs upon adhering to the root surfaces may penetrate through the cell wall and/or transport between cells via plasmodesmata and to aboveground parts via xylem (Iram et al. 2023). Larger-sized *n*CuO may have lower toxicity due to decreased surface reactivity, whereas the smaller-sized *n*CuO (25 nm) that may overcome cellular barriers efficiently may have led to reduced chlorophyll, seed yield, and nutrient quality/yield in soybean. *n*CuO toxicity may not be solely related to Cu^2+^ ions released, as Cu^2+^ ions alone treatments were found to be less toxic than nCuO treatments. Although *n*CuO-25 nm amendment of soil improved micronutrient Cu concentrations in soybean seed, through this work, we further extended our understanding by documenting inhibitory effects on protein, oil, P, K, and seed ultrastructure albeit at a high concentration of 500 mg Cu/kg-soil.

While soil Cu deficiency is a critical problem impacting human health, organic soil areas in California, Oregon, Florida, and Great Lakes in the United States are known to have higher levels of Cu (as high as 495 mg/kg-soil) (Holmgren et al. 1993). In the farmland of mainland China, elevated soil Cu concentrations up to 515.9 mg/kg-soil have been reported (Niu et al. 2013), while in other parts of the world soil Cu concentrations as high as 2000 mg/kg-soil have been documented (Ure and Berrow 1982). Considering that Cu-based NPs can also naturally form in Cu-rich soils via various pedogenic processes, including reactions occurring at soil-root interface (Manceau et al. 2008) and microbial-mediated soil mineralization (Xu et al. 2023), the results of our study employing higher concentration of *n*CuO and Cu^2+^ ions lend credence to deciphering potential toxicity in a major oil crop, soybean, and its underlying mechanism. While NP physicochemical properties and soil types may dictate *n*CuO and Cu^2+^ ions fate in the soil-root interface (Sekine et al. 2017), future research should investigate potential fate of varied size *n*CuO with a focus on speciation at the soil-root interface, within the root, and edible parts such as seed, which will guide health risk assessment of *n*CuO.

## Conclusions

The pursuit of improved sustainable food production has significantly heightened the emphasis on the utilization of nano-fertilizers. However, in order to fully harness the potential of these novel fertilizers, a better understanding of nanophytotoxicity and the intricate underlying mechanisms is warranted. In this study, we show particle size-dependent effects of *n*CuO on the photosynthetic pigments and seed yield and nutrient quality (i.e., protein, oil, P, and K) in soil-grown soybean for a full life cycle of 120 days. Our findings suggest particle size-dependent Cu bioaccumulation-mediated oxidative stress as a mechanism of nCuO toxicity. Future research investigating potential fate of varied size *n*CuO, with a focus on speciation at the soil-root interface, within the root, and edible parts such as seed, will guide health risk assessment of *n*CuO.

### Supplementary Information

Below is the link to the electronic supplementary material.

Supplementary file1 (DOCX 729 KB)

## Data Availability

The data that support the findings of this study are available on request from the corresponding author, SF.
