# Supplementary material for: Role of particle size-dependent copper bioaccumulation-mediated oxidative stress on Glycine max (L.) yield parameters with soil-applied copper oxide nanoparticles
Source: Environ Sci Pollut Res Int. 2024 Apr 2;31(20):28905–21. doi: 10.1007/s11356-024-33070-x (PMC11058571; doi:10.1007/s11356-024-33070-x)
Supplement: Supplementary file 1 — Supplementary file1 (DOCX 729 KB) [file 11356_2024_33070_MOESM1_ESM.docx]

**Supplementary Information**

**Role of particle size-dependent copper bioaccumulation-mediated oxidative stress on *Glycine max* (L.) yield parameters with soil-applied copper oxide nanoparticles**

Elham Yusefi-Tanha^1^; Sina Fallah^1*^; Lok Raj Pokhrel^2*^; Ali Rostamnejadi^3^

^1^Department of Agronomy, Faculty of Agriculture, Shahrekord University, Shahrekord, Iran.

^2^Department of Public Health, The Brody School of Medicine, East Carolina University, Greenville, NC, USA.

^3^Faculty of Electromagnetics, Malek Ashtar University of Technology, Iran.

*Corresponding authors: LR Pokhrel, Phone: 252-737-5587; Email: [pokhrell18@ecu.edu](mailto:pokhrell18@ecu.edu)

S Fallah, Phone: +983832324428; Email: [fallah-s@sku.ac.ir](mailto:fallah-s@sku.ac.ir)


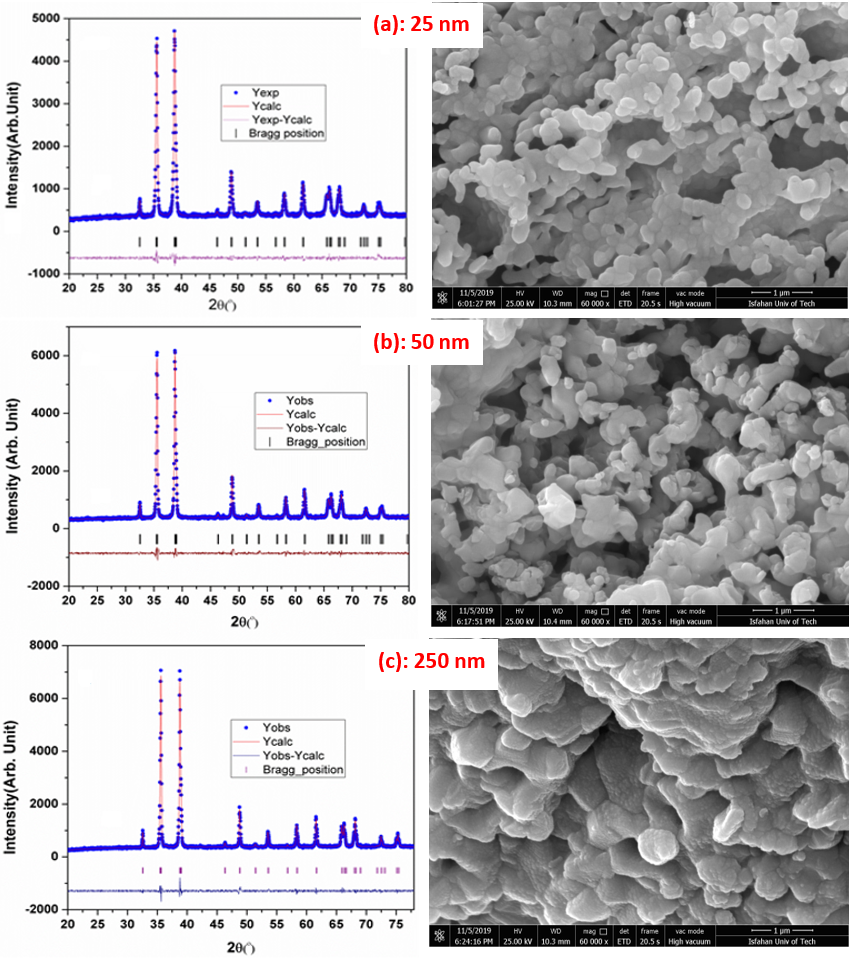
**Figure S1.** XRD patterns with Rietveld analysis and Electron micrographs (FE-SEM) of three discrete size *n*CuO: (a) *n*CuO-25nm = *n*CuO-S; (b) *n*CuO-50nm = *n*CuO-M; and (c) *n*CuO-250nm = *n*CuO-L.

| **Table S1. Analysis of variance (ANOVA) for the effect of copper compound types on Cu accumulation in root and seed, H_2_O_2_ production and malondialdehyde in soil-grown soybean.** | | | | | | | |
| --- | --- | --- | --- | --- | --- | --- | --- |
|  | Mean squares | | | | | df | Source of variation |
| Malondialdehyde | | H_2_O_2_ production | Seed Cu | Root Cu |  | |  |
| 0.0013^**^ | | 107685^**^ | 4.06^**^ | 145.58^**^ | 4 | | Copper compound types |
| 15×10^-6^ | | 344.3 | 0.024 | 0.144 | 10 | | Error |
| 9.47 | | 7.16 | 2.79 | 1.93 | - | | CV (%) |
| **** significant at *p* < 0.01. CV, coefficient of variation.** | | | | | | | |

| **Table S2. Analysis of variance (ANOVA) for the effect of copper compound types on photosynthetic pigments, pod and seed yield in soil-grown soybean.** | | | | | | | |
| --- | --- | --- | --- | --- | --- | --- | --- |
|  | Mean squares | | | | | df | Source of variation |
| Seed production | Seed/pod | Pod/plant | Carotenoids | Chlorophyll-*b* | Chlorophyll-*a* |  |  |
| 34.26^**^ | 0.0026^ns^ | 50.92^**^ | 0.012^**^ | 0.032^**^ | 0.069^**^ | 4 | Copper compound types |
| 0.54 | 0.0066 | 1.27 | 18×10^-5^ | 44×10^-5^ | 65×10^-5^ | 10 | Error |
| 5.23 | 2.74 | 2.83 | 6.34 | 10.12 | 9.81 | - | CV (%) |
| **^ns^ and **, non-significant and significant at *p* <0.01. CV, coefficient of variation.** | | | | | | | |

| **Table S3. Analysis of variance (ANOVA) for the effect of copper compound types on key seed parameters in soil-grown soybean.** | | | | | | | |
| --- | --- | --- | --- | --- | --- | --- | --- |
| Mean squares | | | | | | df | Source of variation |
| Oil yield | Protein yield | Oil content | Protein content | K accumulation | P accumulation |  |  |
| 18923^**^ | 61913^**^ | 1.89^**^ | 47.90^**^ | 0.11^**^ | 0.0021^**^ | 4 | Copper compound types |
| 247.03 | 495.6 | 0.07 | 0.54 | 78×10^-5^ | 92×10^-7^ | 10 | Error |
| 5.61 | 6.62 | 1.34 | 3.18 | 2.27 | 2.63 | - | CV (%) |
| ****, significant at *p* < 0.01. CV, coefficient of variation.** | | | | | | | |

| **Table S4. Analysis of variance (ANOVA) for the effect of copper compound types on antioxidant enzymes activity in soil-grown soybean.** | | | | | | | |
| --- | --- | --- | --- | --- | --- | --- | --- |
|  | Mean squares | | | | | df | Source of variation |
| APX | | POX | CAT | SOD |  | |  |
| 15×10^-5**^ | | 70×10^-6**^ | 70×10^-6**^ | 1.16^**^ | 4 | | Copper compound types |
| 94×10^-8^ | | 96×10^-8^ | 92×10^-8^ | 0.026 | 10 | | Error |
| 6.80 | | 10.60 | 9.51 | 12.78 | - | | CV (%) |
| **** significant at *p* < 0.01. CV, coefficient of variation.** | | | | | | | |
